# Defining the Geographical Range of the *Plasmodium knowlesi* Reservoir

**DOI:** 10.1371/journal.pntd.0002780

**Published:** 2014-03-27

**Authors:** Catherine L. Moyes, Andrew J. Henry, Nick Golding, Zhi Huang, Balbir Singh, J. Kevin Baird, Paul N. Newton, Michael Huffman, Kirsten A. Duda, Chris J. Drakeley, Iqbal R. F. Elyazar, Nicholas M. Anstey, Qijun Chen, Zinta Zommers, Samir Bhatt, Peter W. Gething, Simon I. Hay

**Affiliations:** 1 Spatial Ecology and Epidemiology Group, Department of Zoology, University of Oxford, Oxford, United Kingdom; 2 Malaria Research Centre, Universiti Malaysia Sarawak, Kuching, Sarawak, Malaysia; 3 Eijkman-Oxford Clinical Research Unit, Jakarta, Indonesia; 4 Centre for Tropical Medicine, University of Oxford, Oxford, United Kingdom; 5 Lao-Oxford-Mahosot Hospital-Wellcome Trust Research Unit, Microbiology Laboratory, Mahosot Hospital, Vientiane, Lao PDR; 6 Primate Research Institute, Kyoto University, Inuyama, Aichi, Japan; 7 Department of Immunology and Infection, Faculty of Infectious and Tropical Diseases, London School of Hygiene and Tropical Medicine, London, United Kingdom; 8 Global and Tropical Health Division, Menzies School of Health Research, Darwin, Northern Territory, Australia; 9 Institute of Pathogen Biology, Chinese Academy of Medical Sciences, Beijing, China; 10 Key Laboratory of Zoonosis, Jilin University, Changchun, China; 11 Division of Early Warning and Assessment, United Nations Environment Programme, Nairobi, Kenya; 12 Fogarty International Center, National Institutes of Health, Bethesda, Maryland, United States of America; Arizona State University, United States of America

## Abstract

**Background:**

The simian malaria parasite, *Plasmodium knowlesi*, can cause severe and fatal disease in humans yet it is rarely included in routine public health reporting systems for malaria and its geographical range is largely unknown. Because malaria caused by *P. knowlesi* is a truly neglected tropical disease, there are substantial obstacles to defining the geographical extent and risk of this disease. Information is required on the occurrence of human cases in different locations, on which non-human primates host this parasite and on which vectors are able to transmit it to humans. We undertook a systematic review and ranked the existing evidence, at a subnational spatial scale, to investigate the potential geographical range of the parasite reservoir capable of infecting humans.

**Methodology/Principal Findings:**

After reviewing the published literature we identified potential host and vector species and ranked these based on how informative they are for the presence of an infectious parasite reservoir, based on current evidence. We collated spatial data on parasite occurrence and the ranges of the identified host and vector species. The ranked spatial data allowed us to assign an evidence score to 475 subnational areas in 19 countries and we present the results on a map of the Southeast and South Asia region.

**Conclusions/Significance:**

We have ranked subnational areas within the potential disease range according to evidence for presence of a disease risk to humans, providing geographical evidence to support decisions on prevention, management and prophylaxis. This work also highlights the unknown risk status of large parts of the region. Within this unknown category, our map identifies which areas have most evidence for the potential to support an infectious reservoir and are therefore a priority for further investigation. Furthermore we identify geographical areas where further investigation of putative host and vector species would be highly informative for the region-wide assessment.

## Author Summary

*Plasmodium knowlesi* is a malaria parasite found in monkeys which can infect humans via mosquito bites. People infected with the *P. knowlesi* parasite can suffer severe disease and death yet this disease has often been misdiagnosed as a different malaria type and its geographical distribution is largely unknown. The lack of data on human infections in much of Southeast Asia means a simple map of reported cases would likely misrepresent the extent of the disease. Instead we evaluated and ranked a range of evidence types according to how informative they are about the presence of an infection risk to humans and we mapped this ranked information. This highlighted those geographical areas where new data on the monkey and mosquito species involved in the infection of humans would add most to our knowledge of the full range of factors involved in disease risk. The resulting map highlights known locations of the parasite, and areas where presence of the disease in humans is unknown but possible.

## Introduction

The *Plasmodium knowlesi* parasite, found in wild monkey populations, is a serious public health concern yet almost nothing is known about its geographical extent. It is known to cause severe and fatal disease in humans [1]–[4] and is the most common cause of clinical malaria in high transmission regions of Malaysia [5], [6] where it is three times more likely to cause severe malaria than *P. falciparum* [4]. However, costly *P. knowlesi*-specific molecular diagnostic techniques are only used to confirm diagnosis by microscopy in one area, Malaysian Borneo, whereas human cases have been reported from Brunei [7], [8], Cambodia [9], Indonesia [10], [11], Myanmar [12]–[14], the Andaman and Nicobar Islands of India [15], the Philippines [16], [17], Singapore [18]–[20], Thailand [12], [21]–[24] and Viet Nam [25], [26] as well as most parts of Malaysia [2]–[4], [6], [27]–[42]. The geographical limits of this disease and the spatial variation in disease risk within these limits are simply unknown.

Malaria caused by *P. knowlesi* is a truly neglected tropical disease and there are substantial obstacles to defining the geographical extent and risk of this disease. The symptoms of the disease in humans overlap with those caused by other malaria parasites [43]and other diseases such as dengue [19]. Microscopy fails to distinguish *P. knowlesi* from *P. malariae* (a more benign infection) and *P. falciparum* (the leading cause of severe malaria globally) and in routine practice *P. knowlesi* is also misdiagnosed as *P. vivax* [44], [45]. Currently, Rapid Diagnostic Tests are not only insufficiently sensitive for *P. knowlesi* [46] but can misidentify this species as *P. falciparum* or *P. vivax* (summarised in [43]), and one set of primers used in molecular assays can mistake some *P. vivax* isolates for *P. knowlesi* [47]. The use of routine microscopy has led to large numbers of *P. knowlesi* cases being missed and the parasite is only correctly diagnosed when costly *P. knowlesi*-specific molecular techniques are used. Despite high rates of infection in parts of Malaysia and strong evidence from laboratory experiments that human-to-human transmission by mosquitoes is possible [48], [49], this transmission route is very difficult to demonstrate in nature and to-date no naturally occurring human cases have been definitively linked to human-to-human transmission [43], but equally no barriers to natural human-to-human transmission have been demonstrated.

In the absence of complete geographical data on this disease in humans, the presence of alternative hosts is a useful indicator of the potential presence of a disease reservoir. A competent anopheline vector species is also required for transmission from monkeys to humans (or from humans to humans). These two factors provide an opportunity to map the potential reservoir of the parasite in the absence of human case data. Defining areas of risk, however, is further complicated by the fact that much of the potential parasite range is spread over a large archipelago of many thousands of islands separated by substantial distances; a biogeographical factor often neglected in global disease mapping exercises and of particular relevance to a zoonotic vector-borne disease with a reservoir in wild mammal populations.

Previous studies have defined a range for neglected diseases such as dengue by reviewing the consensus of evidence for the presence/absence of the disease at each location [50]. These studies combined multiple reports of disease presence/absence and weighted them for diagnostic quality and reporting provenance. In the case of *P. knowlesi* malaria, however, there is insufficient direct evidence of disease presence/absence to replicate this approach. In this study, instead of assessing the consensus of evidence for disease presence/absence, evidence on locations of host and vector species, as well as human case data, were combined to obtain ranked scores for the capacity to support an infectious reservoir. We first reviewed the evidence on non-human primate hosts and transmission by different anopheline vector species and then gathered data on the ranges of these two groups, as well as the locations of known human cases of the disease. This information was used to assess the potential of each province or island to support an infectious reservoir. The final output is a comprehensive summary of the current state of evidence for a *P. knowlesi* reservoir. Importantly, it is not a map of the likelihood of a reservoir occurring within an area but it does highlight areas where evidence is lacking. The results of this study allow us to propose priorities for the new data that are urgently needed in order to understand the spatial variation in risk to humans from this disease.

## Methods

### Defining the area of study

Maps of human disease often use administrative divisions to subdivide countries. This is the structure in which much national health data are provided and is a useful format to feed results back to public health agencies. For zoonotic diseases, however, the distributions of wild host species will not necessarily map closely to administrative divisions. In this instance, the majority of cases reported to-date are located within a huge archipelago where administrative divisions can encompass multiple islands separated by large distances. For this study we took a mixed approach using administrative divisions to subdivide the mainland and the largest islands in the archipelago (Papua, Borneo, Sumatra, Java and Sulawesi). The largest administrative division in the area of study, Xizang Zizhiqu (the Tibet Autonomous Region) in China, was further divided into level two divisions. Additionally, islands greater than 25 km from the mainland and greater than 200 km^2^ in area were defined as separate geographical units. Within the archipelago, islands within 10 km of each other were grouped together and islands less than 100 km^2^ and more than 10 km away from any other island were disregarded. Following these approximate guidelines we were able to divide this region of 19 countries spread over approximately 25,000 islands into 475 geographical units.

### Reviewing the evidence for the pre-requisites required to support a *P. knowlesi* reservoir

We conducted a literature survey in Web of Knowledge using the terms ‘knowlesi’, ‘zoono* and malaria’, ‘monkey and malaria’ to collate journal articles on the parasite and then excluded studies conducted solely in the laboratory (e.g. immunity studies using a rhesus-knowlesi model). The bibliography of each article was then searched for further published sources of information and authors working in locations of particular interest were contacted. The search was completed on 30 September 2013. Molecular techniques that can distinguish the *P. knowlesi* parasite (alone or in combination with microscopy) have only been available for the last decade so this dictated the period reviewed (2004 to 2013). All data on wild animals tested for *P. knowlesi* infection were extracted and used to determine which alternative host and vector species would contribute to the next stage of the work.

### Ranking the evidence for presence of a *P. knowlesi* reservoir infectious to humans, by subnational area

**Figure 1:**
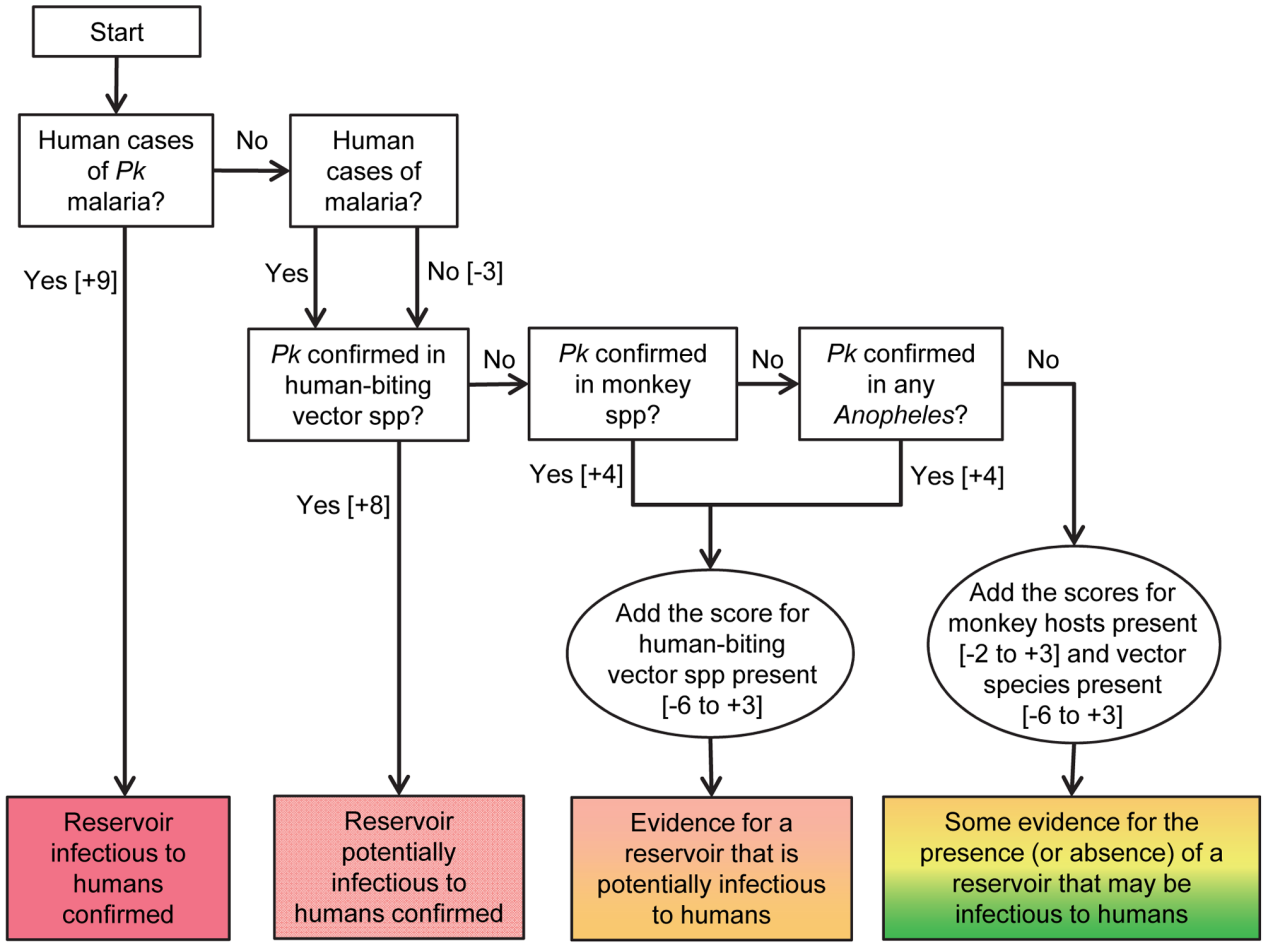
This schematic outlines the system used to assign an evidence score to each area. Further details are provided in the text. *Pk* = *P. knowlesi*.

Each subnational area was assigned a score based on three classes of evidence: the presence of the parasite; the presence of a monkey host species, and; the presence of a malaria vector species known to bite humans (Figure 1). Each area was scored independently and was unaffected by the scores of neighbouring areas to reflect, in part, the patchy nature of the disease and of the evidence.

The scores assigned provide a simple ranking. In summary, confirmation of a human infection ranked highest with +9 overriding all other evidence, then confirmation of sporozoites in a human-biting vector scored +8. Confirmation of a monkey infection was combined with the score for presence of a human malaria vector (see below) to give a maximum score of +7. In the absence of the parasite itself, presence of both a known host species and a known vector species scored +6. Combinations of known/putative host species presence and known/putative vector species presence (see below) scored from +2 to +5. Combinations of host species absence, vectors species absence and absence of any malaria in humans (see below) scored values ranging from −1 to −9. In locations where both presence and absence indicators were found, the respective scores were added together.

### Scoring the evidence for *P. knowlesi* presence and other human malaria parasites

All occurrences of the parasite, identified using either 1) *P. knowlesi*-specific molecular identification methods or 2) a combination of microscopy and molecular techniques that distinguish *P. knowlesi* from *P. falciparum* and *P. malariae*, were extracted from the library of published literature described above. The location of occurrence was defined as the location of infection, not the location of symptom onset or diagnosis, and studies that could not identify the location of infection (to state/island level) were excluded. For each occurrence, the date of study, diagnostic technique(s) and subnational location of infection were extracted. Only the most recent infection from each area/island was retained. In two studies we could not distinguish between adjacent administrative divisions so these areas were combined (at the Myanmar/China border and at the Myanmar/Thailand border). Two reports of human cases from Brunei did not meet the inclusion criteria because one used microscopy only for diagnosis [8] and the other did not publish their diagnostic methods [7].

There were no survey results that provided clear evidence for absence of the parasite in an area. In countries that routinely report cases of the four human malarias, occurrence of these other species may mask *P. knowlesi* cases and, conversely, divisions within these countries that report no malaria cases are less likely to have undetected *P. knowlesi* cases. Areas within malaria endemic countries reporting no malaria cases were defined using the 2012 World Malaria Report [51] and assigned a score of −3. For areas without data in the 2012 World Malaria Report, we used the 2010 limits of *P. falciparum* and *P. vivax* defined by the Malaria Atlas Project to classify each area [52], [53].

### Scoring the evidence for alternative host (monkey) presence

Based on the data collected from published studies (see Results), we made the decision to use the ranges of two monkey species. *Macaca fascicularis* (the long-tailed or crab-eating macaque, also known as the cynomolgus or kra monkey) and *M. nemestrina* (the pig-tailed or Southern pig-tailed macaque). Two other species have been identified as hosts, *Trachypithecus obscuras* and *Presbytis melalophus*, however the ranges of these species fall entirely within the range of *M. fascicularis*, therefore, these areas already receive the maximum score for presence of a known non-human host species.The ranges for *M. fascicularis* and *M. nemestrina* were initially defined using the International Union for Conservation of Nature (IUCN) ranges [54] and a score of +3 assigned to all subnational areas that overlapped with one or both of these ranges. The IUCN ranges, however, estimate the natural range of each species and do not always include introduced populations, and new data may have been collected since the ranges were last revised. For these reasons we also included evidence for presence of each species outside the IUCN ranges from 1985 onwards and gave a score of +3 to records of a host species collected since 2000 and +2 for records collected between 1985 and 1999. A score of +1 was assigned to areas where the published evidence indicated introduced populations have hybridised out with endemic species in the area. The published literature does not cover all islands in the Malay Archipelago so we also contacted conservation and wildlife organisations in Indonesia, Malaysia and the Philippines to request information on which islands support populations of these species and assigned a score of +3 to any new areas identified by these organisations. After published studies reported finding *P. knowlesi* infections in *M. nemestrina* monkeys, this monkey species was divided by taxonomists into *M. nemestrina* and *M. leonina* (the Northern pig-tailed macaque). We made the decision to include both species because, although it is likely that the monkeys tested were *M. nemestrina* (as currently classified), human cases have been found outside the ranges of *M. nemestrina* and *M. fascicularis* but within the range of *M. leonina*. A lower score of +2 was assigned to areas within the *M. leonina* range.

The IUCN ranges were combined for all three macaque species and a score of −1 was assigned to areas outside the combined host species range (excluding locations with introduced populations) and −2 for those areas more than 100 km outside this range. The maximum possible negative score was not assigned because we do not have a definitive list of primate species that can host a reservoir of *P. knowlesi* parasites in the wild, and laboratory studies have shown that other species can be infected by this parasite [55].

### Scoring the evidence for anopheline vector presence

Based on evidence from the published literature on which *Anopheles* species are capable of transmitting *P. knowlesi* (see Results) and evidence for which vectors transmit human malaria [56], we assigned the highest vector score of +3 to the Leucosphyrus Complex and the Dirus Complex. This score was assigned to areas where a human malaria vector belonging to either of these two Complexes was recorded as present. Specifically we used published ranges for the Dirus Complex, *Anopheles leucosphyrus* and *An. latens* combined, and *An. balabacensis*. The species were grouped in this way because studies publishing vector species occurrence frequently do not distinguish individual species within these groupings. In the absence of these species, the presence of other sylvatic vector species (forest/margins dwelling, and therefore more likely to encounter macaques) known to transmit malaria to humans but of unknown *P. knowlesi* vector status was assigned a lower score of +2. The species in this category were the Fluviatilis Complex, the Minimus Complex, *An. koliensis*, *An. aconitus*, *An. annularis*, the Culicifacies Complex and *An. flavirostris*. Finally, where no vector species from either of the above two classes were present, presence of any of the other human malaria vectors was assigned a lower score of +1 to reflect the fact that these species are known to have the capacity to transmit malaria parasites to humans [56] and have not been ruled out as vectors of *P. knowlesi*. To assess the presence of all three vector classes, we used the predicted distributions generated by the Malaria Atlas Project [56] and defined all points with a probability of occurrence of >0.5 as presence locations. Presence of any one of the species from a vector class within an administrative division or island was considered sufficient to record that vector class as present.

A score of −4 was assigned to areas outside the combined range of the vector species and −6 to areas 100 km outside this range. This score (smaller than the maximum negative score but greater than the negative score assigned to absence of known monkey host species) reflected the fact that there is a lack of evidence for the definitive list of vectors transmitting *P. knowlesi* but much stronger evidence for the definitive list of vectors that transmit malaria to humans.

### Calculating the overall score

The scores were combined as shown in Figure 1 and the overall scores, providing a relative ranking of the cumulative evidence for each subnational area, were displayed on a map of the region. A second simplified map was then created, to aid visualisation of the results, by grouping the scores into four classes: scores of +7 to +9 were classed as ‘confirmed infectious reservoir’; scores of +6 were classed as ‘confirmed reservoir prerequisites’; scores of +1 to +5 were classed as ‘weak evidence for a reservoir’; and scores of −9 to 0 were classed as ‘absence of reservoir prerequisites’.

To test the scores generated, the scores that would have been obtained if evidence for presence of the parasite itself was excluded were compared between areas with confirmed parasite presence and those of unknown parasite status. A jackknife approach was then used to assess the dependence of the final scores on each individual factor. Each individual factor was excluded and the scores were re-calculated. The results were compared between areas with confirmed parasite presence and those of unknown parasite status, and the relative ranking of all areas before and after each factor was removed were compared. To assess the predictive power of the scores, the area under the receiver operating characteristic curve (AUC) was calculated for each version of the scoring system created when single factors were removed in turn (with parasite presence excluded) [57].

## Results

The review of published studies of *P. knowlesi* infection in wild monkey populations is summarised in Table 1. It is immediately clear that only a few species and populations have been tested in a few countries. High infection prevalences have been found in *M. fascicularis* and *M. nemestrina* populations in Sarawak in Malaysian Borneo and lower prevalences in Singapore, Kuala Lumpur and Pahang States in Malaysia, Narathiwat and Ranong Provinces in Thailand, and North Sulawesi Province in Indonesia. Older studies (pre-2004) have also found infected *M. fascicularis* monkeys in Cebu, Philippines [58].

**Table 1:** Published cases of *P. knowlesi* infection in non-human primates, from studies conducted since 2004.

	No. individual monkeys positive for *P. knowlesi* infection/no. tested					Ref.
	*M. fascicularis*	*M. nemestrina* *	*P. melalophus*	*T. obscurus*	Other species	
**Bangladesh**						
**Bhutan**						
**Brunei**						
**Cambodia**						
**China**						
**India**						
**Indonesia**	1/31					[70]
**Laos**						
**Malaysia**	10/143	0/1	0/1			[29]
	71/82	13/26				[86]
**Myanmar**						
**Nepal**						
**Palau**						
**PNG**						
**Philippines**						
**Singapore**	3/13					[18]
**Sri Lanka**						
**Thailand**	0/99					[87]
	1/195	4/449		1/7	0/4	[88]
**Timor Leste**						
**Vietnam**						
**TOTAL**	**86/563**	**17/476**		**1/7**	**0/4**	

* *Macaca nemestrina* has since been divided into sibling species *M. nemestrina* and *M. leonina*.

The review of published studies of *P. knowlesi* in wild mosquito populations is summarised in Table 2. The most striking result is that published studies have only been conducted in Khanh Hoa Province in Vietnam and Pahang State and Kapit Division of Sarawak State in Malaysia. Other countries have very different vectors that are known to transmit malaria to humans but their role in *P. knowlesi* transmission is unknown. In the areas studied, there is evidence that *Anopheles latens* from the Leucosphyrus Complex and members of the Dirus Complex transmit *P. knowlesi*. Members of both Complexes are known to transmit human malarias. Earlier studies (pre-2004) have implicated members of the Hackeri Subgroup in transmission of *P. knowlesi* within monkey populations in Peninsular Malaysia [59], however, these mosquito species are not known to bite humans. Laboratory studies have shown that a wider range of species may be able to transmit *P. knowlesi*, however, these studies also confirmed that the most effective vectors, of those tested, were members of the Leucosphyrus Group [60], [61].

**Table 2:** Published cases of *P. knowlesi* infection in *Anopheles* vectors, from studies conducted since 2004.

	No. individual mosquitoes positive for *P. knowlesi* infection/no. tested							Ref.
	*An. latens*	*An. introlatus*	Dirus Complex	Hackeri Subgroup	Riparis Subgroup	Other Leucosphyrus Group	Other species	
**Bangladesh**								
**Brunei**								
**Cambodia**								
**China**								
**India**								
**Indonesia**								
**Laos**								
**Malaysia**			2/211	0/1			0/127	[29]
	4/1073	0/4		0/9	0/8		0/1414	[89]
	8/339							[90]
			4/940	0/2		0/5	0/540	[91]
**Myanmar**								
**Nepal**								
**Palau**								
**PNG**								
**Philippines**								
**Singapore**								
**Sri Lanka**								
**Thailand**								
**Timor Leste**								
**Vietnam**			37/5686					[26]
**TOTAL**	**12/1412**	**0/4**	**43/6837**	**0/12**	**0/8**	**0/5**	**0/2081**	

**Figure 2:**
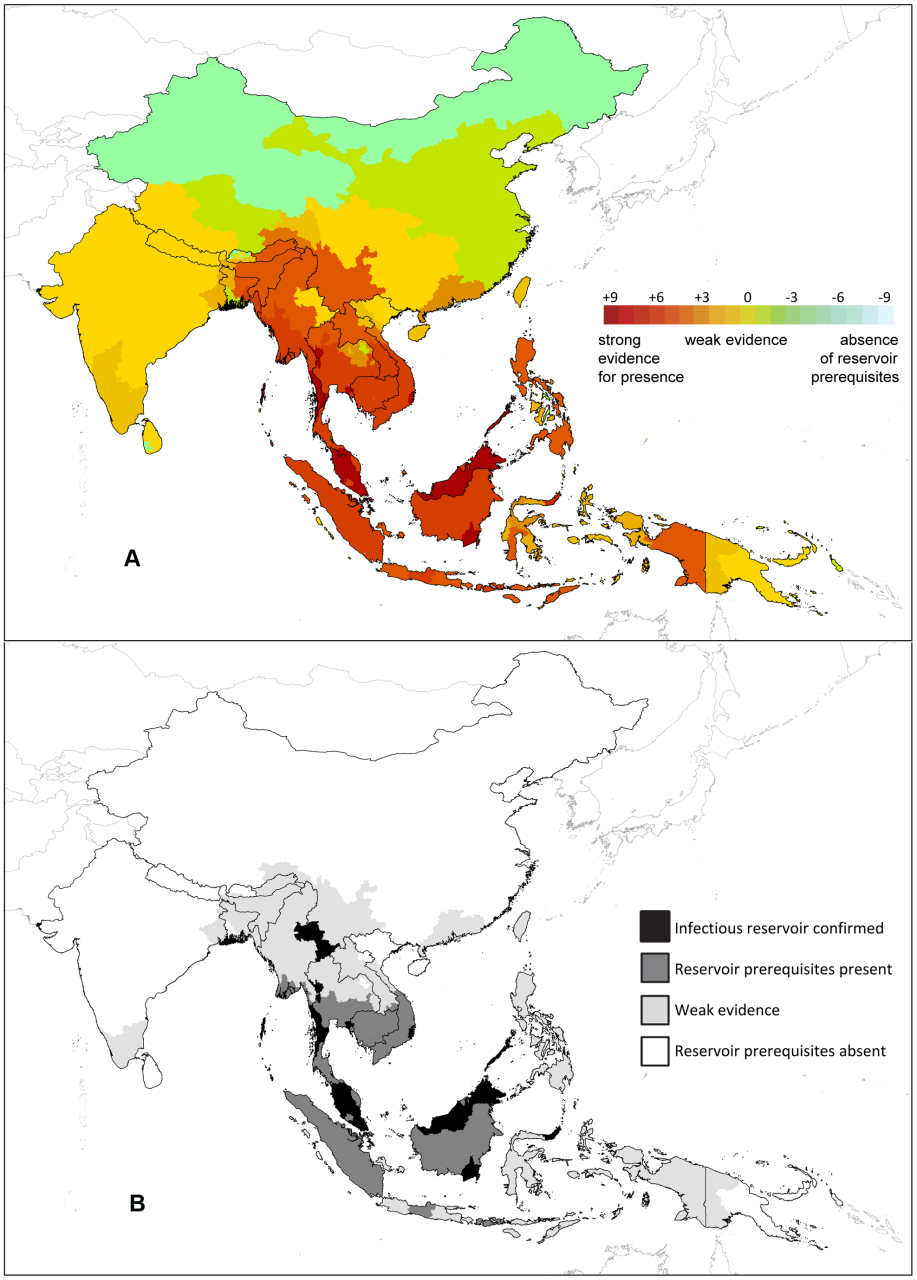
Panel A is a map displaying the evidence scores assigned to each area ranging from strong evidence for presence of a parasite reservoir infecting humans to weak or no evidence through to absence of host and vector species indicating that an infectious reservoir would not be supported. Panel B shows the same scores grouped into four classes.

The information from the reviews of monkey hosts and of vectors was used to generate parasite, host and vector evidence scores for each geographical area and these were combined with the evidence for parasite presence to give an overall score representing the evidence for potential presence of a parasite reservoir that is infectious to humans (shown in Figure 2A). The individual evidence scores assigned to each subnational area (for evidence of human infection, parasite occurrence, known and potential host occurrence, and known and potential vector occurrence) are given in Table S1.

Figure 2A shows the full range of scores generated. The variation in cumulative evidence for presence of the prerequisites required to support an infectious reservoir can be seen, from a complete absence of all prerequisites and thus evidence for absence of a reservoir (−9,) to a lack of evidence and high uncertainty (0), to presence of a full set of prerequisites but unknown parasite status (+6), to confirmation of human cases (+9). Figure 2B shows a simplified version of the same information with the scores grouped into four classes: areas where both the parasite itself and a vector able to transmit it to humans have been found; areas with known monkey hosts, known vectors of *P. knowlesi* and no factors indicating absence of a reservoir (presence of the parasite itself is unknown); areas of weak evidence for the presence of a full set of reservoir prerequisites; and areas where there is evidence for an absence of reservoir prerequisites. It is important to note that Figure 2 is not a map of the likelihood of a reservoir occurring within each area, for example, an area may receive a zero score because evidence is lacking or it may in fact be less likely to support an infectious reservoir.

**Figure 3:**
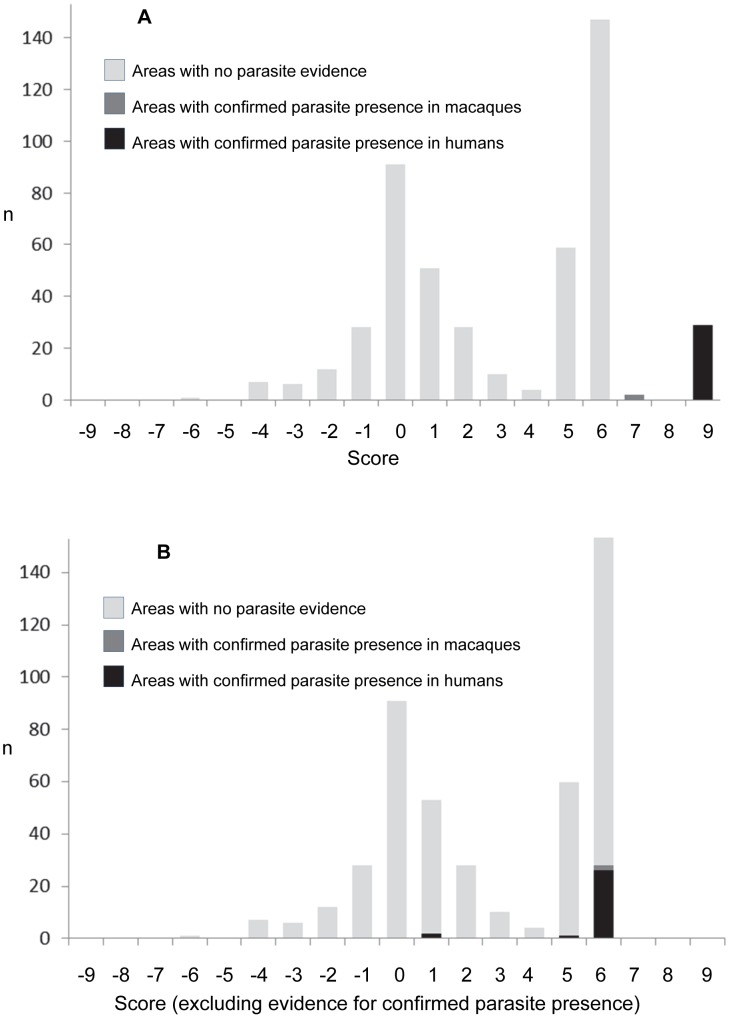
Panel A shows the distribution of scores for each subnational area. Panel B shows the scores assigned when the evidence for parasite presence was removed (i.e. the scores based solely on host presence/absence, vector presence/absence, other human malaria presence and presence of *M. mulatta*). Those areas with confirmed parasite presence are shown in black.

Figure 3A provides a histogram of the full range of scores assigned to the 475 subnational areas with scores +7 to +9 exclusively assigned to areas with confirmed parasite presence. Figure 3B shows the range of scores assigned when evidence for parasite presence was excluded from the scoring system. The scores assigned to areas that are known to support the parasite ranged from +1 to +6, i.e. the parasite has been found in areas outside the known monkey and/or vector ranges, or areas with factors that indicate absence of a reservoir prerequisite. The area that scored +5 was the northern part of Myanmar (Shan State North and East) bordering China, and the evidence for parasite presence here came from two independent studies [13], [14]. The known monkey host species (*M. fascicularis* and *M. nemestrina*) have not been found in this area but *M. leonina* is present. Studies that have investigated malaria parasites in the monkey populations in this area have not yet found evidence of *P. knowlesi* infection in any of the species present (Qijun Chen, unpublished data).

Two neighbouring areas with confirmed parasite presence scored only +1 when the evidence for parasite presence itself was excluded. These were two islands in the north of the Andaman and Nicobar Islands; Smith Island and Car Nicobar. The southern islands fall within the range of *M. fascicularis* but there is no evidence of any known or putative monkey host species populations on the northern islands, including Smith Island and Car Nicobar [62]. The evidence for human *P. knowlesi* infections in these locations (and also on Great Nicobar and Teressa, two of the southern islands with known hosts and known vectors) comes from a single study of human malaria cases in the Andaman and Nicobar Islands [15]. A total of 15 cases were found on Smith Island and 25 on Car Nicobar, which rules out a one-off imported case. Further work is required to investigate the possibility of a *P. knowlesi* reservoir existing on the northern islands of the Andaman and Nicobar Islands, including the possibility of human-to-human transmission and the possibility of a parasite reservoir in the captive long-tailed macaques at Port Blair's zoo [63].

Finally, Figure 2 shows that many areas in the region have weak evidence for their ability to support an infectious reservoir, but cannot be ruled out altogether. A small number of areas fall outside the ranges of all known or putative hosts or vectors, which provides evidence that these areas could not support a *P. knowlesi* reservoir. If this study had covered a broader geographic area, the number of these areas would be much higher.

Table 3 provides the AUC values calculated when evidence for parasite presence was excluded, and each time the scoring system was adjusted to remove a single factor in turn. The AUC value obtained when parasite presence only is excluded was 0.7979, indicating the scoring system has very good predictive power. No set of factors modelled the known locations of the parasite perfectly but the result was similar when different factors were removed and was always ≫0.5, indicating that the accuracy of the scoring system is always good and not heavily dependent on any single factor. Figure S1 shows the full range of scores obtained when individual factors were excluded. In each case, scores for the subnational areas with confirmed parasite presence (31 subnational areas; 29 with confirmed human cases and 2 confirmed in monkey only) can be visualised compared to the scores for the 444 other areas. Figure S2 shows the relative ranking of the 475 areas after a single factor has been removed, against the ranking using all factors. The relative ranking does not appear to be strongly affected by the removal of any single factor, i.e. the individual factors are highly correlated as indicated by the consistently high AUC values shown in Table 3. Table S2 provides the full set of scores for each area including the scores achieved after each individual factor had been removed.

**Table 3:** The area under the curve of the receiver operating characteristic for the overall model excluding evidence for parasite presence and when individual factors were excluded in turn.

Model (scoring system)	Area under the curve (AUC)
*P. knowlesi* presence excluded	0.7979
*P. knowlesi* presence and *Leucosphyrus* vectors excluded	0.7319
*P. knowlesi* presence and other sylvatic vectors excluded	0.8037
*P. knowlesi* presence and other human vectors excluded	0.7990
*P. knowlesi* presence and vector absence excluded	0.7978
*P. knowlesi* presence and other human malarias excluded	0.7875
*P. knowlesi* presence and the natural range of *M. fasciularis* excluded	0.8624
*P. knowlesi* presence and) the natural range of *M. nemestrina* excluded	0.7990
*P. knowlesi* presence and introduced *Mf/Mn* populations excluded	0.7808
*P. knowlesi* presence and *M. leonina* excluded	0.7970
*P. knowlesi* presence and host absence excluded	0.8055

*Mf* = *M. fascicularis* and *Mn* = *M. nemestrina*.

## Discussion

By assessing the evidence in a systematic way based on the current state of knowledge, we have been able to map subnational areas where a *P. knowlesi* reservoir capable of infecting humans has been confirmed and those that support known hosts and vectors. In the absence of routine confirmation of *P. knowlesi* in human cases, and of definitive lists of host and vector species, it is harder to map areas of known disease absence. We have, however, been able to classify the rest of the region into areas that range in the evidence for their capacity to sustain a parasite reservoir that is infectious to humans, based on the current state of knowledge. Both the review of species shown to host and transmit *P. knowlesi*, and the ranking of the evidence for a parasite reservoir, highlight the urgent need for more evidence in large parts of the region of study and provide information on the types of data that are needed. The results of this study highlight priority geographical areas for future study that would enable us to build a more precise map. Areas of Indonesia (Kalimantan, Sumatra, part of Java and parts of Sulawesi), parts of the Philippines, Cambodia, S. Thailand, S. Myanmar and S. Vietnam support both the known hosts and the known vectors, and are obvious targets for studies investigating new locations of parasite infections and disease prevalence. Locations with high disease potential could be targetted further by identifying areas that report cases of *P. malariae* malaria when using microscopy for routine species confirmation. The blank cells in Tables 1 and 2 indicate the regions that have not been tested for parasite presence in alternative hosts and vectors, and the species that have not been tested. In this case, data on absence of the parasite will be as important as presence data and will help to refine the disease limits.

When parasite presence was excluded from the scoring system, the predictive power of the scores generated from the evidence on hosts, vectors and human malarias was very good (AUC = 0.8146). It is important, though, not to assume that the factors used in this scoring system give the full picture. It is likely that the researchers who designed the *P. knowlesi* studies conducted outside of Malaysia used the same assumptions about host and vector species as this study, when choosing their study locations, leading to a bias in locations where the parasite has been found. Evidence from human cases in returning travellers, however, may not be subject to the same biases for presence of presumed host and vector species. All of the published cases of *P. knowlesi* infection in returning travellers, diagnosed outside the region, involve patients that had spent time in one or more subnational areas where both the known monkey hosts and the known vectors are present [7], [10], [17], [22], [30], [31], [34], [35], [37], [64]–[66] providing corroborating evidence for the assumptions made in this study. Absence of a parasite is harder to prove and negative results are harder to publish, but there is a limited amount of unpublished data that provides further corroboration of our approach. Investigation of 349 human malaria cases from across Laos (average score 4.25 = weak evidence) found no *P. knowlesi* (M. Mayxay, unpublished data) while surveys of macaque populations in Nepal (average score −1 = weak evidence/absence of reservoir prerequisites) and Bangladesh (average score 1.67 = weak evidence) also found no evidence of *P. knowlesi* infection (Ananias Escalante, unpublished data).

The sensitivity analysis presented here suggests that some of the factors included in this study could be removed and the scores would still perform as well, however, the areas with confirmed parasite presence are a potentially biased sample and so it would be unwise to remove any potential factors until we are closer to a definitive list of vectors and alternative hosts. This would help to refine the map presented here, enabling us to assign higher positive or negative scores for either presence or absence, and therefore to delineate more accurately the outer limits of the disease reservoir. This is necessary in order to provide precise information to public health agencies, and to provide a contemporary baseline to monitor future changes in the disease distribution. Longitudinal studies in Sabah, Malaysia have shown that *P. knowlesi* incidence has increased at this location over the last decade [6] but further research is required to assess whether this is linked to factors such as changing land use, changes in human behaviour and/or changes in the behaviour of the alternative hosts or vectors, including the possibility that human-to-human transmission is a factor [67].

In the past a lack of diagnostics meant that data on human cases was lacking and high population movement further complicated the picture. We are now in a better position to obtain human case data and this study has highlighted the regions to target. Further studies of the monkey species able to host this parasite would also be particularly informative, particularly in Northern Myanmar where *M. fascicularis* and *M. nemestrina* are absent but *M. leonina*, *M. assamensis* and *T. phayrei* are present [54]. Studies of any monkey populations on Smith Island in the Andamans would also be informative although no monkey species are endemic to this island or nearby Car Nicobar [54] and there are no confirmed reports of domestic or introduced primate species on these islands. Port Blair Zoo on Smith Island has long-tailed macaques in captivity [63] but there is no evidence for presence of captive monkeys on Car Nicobar. The report of human cases of *P. knowlesi* malaria on Smith Island and Car Nicobar [15], and the primate status of these islands, certainly merits further investigation. Both the monkey and vector species involved in *P. knowlesi* transmission are a complex and dynamic mix of subspecies and sibling species [56], [68]. No studies to-date have considered the ability of the full range of macaque species to host malaria parasites nor have the many hybrids occurring in areas where these species are co-endemic [69] been investigated for their parasite status.

As well as their susceptibility to *P. knowlesi*, the social organisation of these primates differs, in terms of ranging patterns, relationships to humans and time spent on the ground versus the canopy. These factors may have an important influence on their relevance as a reservoir for transmission of *P. knowlesi* to humans. These factors also differ between populations; for example, in areas with extensive primate hunting, primate reservoir populations may be pushed far from humans reducing the probability that humans will intersect with primate-vector cycles.

This work also highlights the importance of understanding the role of introduced populations when the ultimate goal is to map a disease reservoir. *Plasmodium knowlesi* has been found on Sulawesi [70] where none of the host species are endemic but where *M. fascicularis* and *M. nemestrina* are kept as pets and have escaped into the wild [71]–[73]. Future studies of parasites in introduced populations would increase our understanding of the likelihood of a founder population being infected and of persistence of the parasite within a new population following introduction. This would inform the criteria used to decide which populations are included in mapping studies.

When monkey host species have been introduced to areas where other macaque species are endemic [74], hybrids have been found outside the range of the host species and further interbreeding may lead to genes from these host species being introgressed into the native species population [75]. This again raises the question of the infection status of hybrids and of non-host species populations with introgressed genes from *M. fasciularis* or *M. nemestrina*. Hybrids are likely to occur in the narrow contact zone between *M. fascicularis* and *M. mulatta* in Thailand, Vietnam, Laos and Myanmar [76], [77]. Hybrids found within the range of known *P. knowlesi* host species will not impact the geographical limits of the disease, but if hybrids are found beyond the range of the known hosts this could affect the disease risk in these locations.

The disease status of hybrids between known hosts, whose populations can sustain high *P. knowlesi* infection prevalences, and rhesus monkeys, that may not be able to survive in the presence of *P. knowlesi*, is particularly interesting and currently unknown. The impact on *P. knowlesi* host status following introgression of genes from one species into populations of another is also unknown; we simply do not know whether introgression (contemporary or ancient) of *M. fascicularis* genes into *M. mulatta* populations at the southern end of their range [78]–[80] increases their ability to host this parasite or whether introgression of genes from *M. mulatta* into *M. fascicularis* populations north of peninsular Thailand reduces their ability to act as a reservoir for this parasite, or whether more complex genetic interactions occur. Which genes are important in *P. knowlesi* infection and their status in any of these species or populations is unknown. Taking a pragmatic approach for the purpose of producing a map of disease risk for public health use, however, the most important questions are 1) what is the prevalence of human infection at precise locations and 2) what is the prevalence of infection in species/hybrids of monkeys and mosquitoes at precise locations across the region? A recent preliminary finding reports a *P. knowlesi* infection in either a *M. mulatta* monkey or a *M. mulatta*/*M. fascicularis* hybrid in Vietnam close to the location of known human and vector infections [81]. Further investigation *of P. knowlesi* in wild *M. mulatta* populations, and in populations of hybrids, is needed to provide evidence for their role in hosting a parasite reservoir.

Large numbers of laboratory experiments have shown that *P. knowlesi* readily infects and is usually fatal to *M. mulatta* (∼70% of individuals are killed) but the surviving animals have the ability to pass the parasite on to mosquito vectors [82], leading to contrasting hypotheses that either *M. mulatta* populations cannot co-exist with the parasite [83] and could therefore be used as a negative indicator for a disease reservoir, or *M. mulatta* could be a natural host for the parasite [81] and therefore be used as a positive indicator. Alternatively, populations of *M. mulatta* in different locations may differ in their level of immunity to *P. knowlesi*, which would mean both hypotheses could be true depending on location. The overlap of the *P. knowlesi* parasite and the rhesus monkey found in two subnational areas (N Myanmar and NW Thailand) suggests the parasite and the rhesus macaque may co-exist, however, within these two areas the precise locations of the parasite and of the macaque may still differ. A finer scale approach will help to resolve the question of whether *M. mulatta* can co-exist with *P. knowlesi*. In addition, a second monkey species, *Presbytis* (*Semnopithecus*) *entellus*, which is also known to have a high fatality rate when infected in the laboratory [84], could be used as an indicator of *P. knowlesi* absence or alternatively this species could also provide a parasite reservoir. Inclusion of *P. entellus* as a negative indicator would reduce the scores for areas of Southern India and Sri Lanka on the edges of the area of this study. More data on the naturally-occurring malaria infections found in the full range of species in the region is needed and further data to back up the finding of a *P. knowlesi* infection in either a *M. mulatta* monkey or a *M. mulatta*/*M. fascicularis* hybrid in Vietnam [81] would resolve the issue of whether *M. mulatta* presence is a useful indicator for a potential disease reservoir.

The approach used in this paper has the limitation that presence of a single isolated host population in one part of a state/island increases the score assigned to the whole state/island. One example of this is Papua, a Province of 320,000 km^2^ which is not endemic for any of the known or potential host species but which supports a single isolated population of *M. fascicularis* near Jayapura [85]. Surveys of the malaria parasites found in isolated introduced populations would provide an evidence base for the score assigned to these populations. Furthermore surveys which show that an isolated population is *P. knowlesi*-free can be used to exclude the population from the scoring system. A surveyed population that is found to be infected will still affect the score for the whole state/island, as will an isolated human case or infected vectors at a single discrete location, and a finer resolution mapping approach is needed to address this limitation of the current map.

The map presented here has divided the region into 475 areas and provides good subnational resolution, but it is not a fine resolution map and cannot distinguish the large variation that may exist within a province or island. Specifically, there will be areas within most provinces/islands that are less likely to support a reservoir. Point-located data for the parasite, hosts and vectors is available, which opens up the possibility of using ecological niche modelling techniques to produce a finer resolution map. Niche models will identify areas suitable for disease transmission that fall outside the actual disease range, unless constrained by information on the geographical extent of the disease. The study published here has used an evidence-based approach to examine the putative range of the disease reservoir and can be used to delineate outputs from studies that use a niche modelling approach to map this disease on a fine scale. Furthermore, the methods developed in this study are broadly applicable and could usefully be extended to other severely neglected vector-borne and/or zoonotic diseases such as scrub typhus or chikungunya.

The goal of the map presented here is to provide a comprehensive summary of the current state of evidence for a *P. knowlesi* reservoir. It is not a map of the likelihood of a reservoir occurring within each area and an area may receive a zero score because the evidence available is lacking or it may in fact be less likely to support an infectious reservoir. This issue is particularly apparent within the Malay Archipelago. Smaller islands are less likely to have evidence for parasite and/or host and/or vector presence, but they may also differ in their underlying ability to support a parasite reservoir. The map shows where evidence is strong and is inherently biased to areas where studies have been conducted. When considering methods to model the probability of occurrence of a reservoir, on a fine-scale, it will be essential to address the issue of sample bias. In order to produce such fine scale maps, more data is needed and the current study has highlighted the types of data and the geographical areas of study that would be most informative, based on our current state of knowledge.

## Supporting Information

Figure S1 A figure showing histograms of the scores generated each time the scoring system was adjusted. Subnational areas with confirmed cases of knowlesi malaria in either humans or macaques are marked in black and all other areas are light grey. Panel A shows the scores when evidence of parasite presence is excluded. Panels B–L show the scores generated when a second individual evidence class is excluded: B) *Leucosphyrus* vectors excluded; C) other sylvatic vectors excluded; D) other human vectors excluded; E) combined vector range excluded; F) other human malarias excluded; G) the natural range of *M. fasciularis* excluded; H) the natural range of *M. nemestrina* excluded; I) introduced *M. fascicularis* and *M. nemestrina* populations excluded; J) *M. leonina* excluded; K) combined monkey range excluded.
(TIF)

Figure S2 A figure showing ranked scores generated when individual factors were excluded. Each graph shows the ranked scores when evidence of parasite presence is excluded (the x axis) against the ranked scores when the score is adjusted as follows: A) the mean score obtained across all exclusions (B–L); B) *Leucosphyrus* vectors excluded; C) other sylvatic vectors excluded; D) other human vectors excluded; E) combined vector range excluded; F) other human malarias excluded; G) the natural range of *M. fasciularis* excluded; H) the natural range of *M. nemestrina* excluded; I) introduced *M. fascicularis* and *M. nemestrina* populations excluded; J) *M. leonina* excluded; K) combined monkey range excluded.
(TIF)

Table S1 An Excel file containing the full set of individual scores for each evidence class and the overall evidence score as displayed in Figure 2A of the manuscript.
(XLSX)

Table S2 An Excel file containing the scores assigned to each subnational area when parasite evidence was excluded and the scores generated when each individual evidence class was removed.
(XLSX)

## References

[1] Cox-Singh J, Hiu J, Lucas SB, Divis PC, Zulkarnaen M, et al (2010) Severe malaria - a case of fatal *Plasmodium knowlesi* infection with post-mortem findings: a case report. Malar J 9: 10. 20064229 10.1186/1475-2875-9-10 PMC2818646

[2] William T, Menon J, Rajahram G, Chan L, Ma G, et al (2011) Severe *Plasmodium knowlesi* malaria in a tertiary care hospital, Sabah, Malaysia. Emerg Infect Dis 17: 1248–1255. 21762579 10.3201/eid.1707.101017 PMC3381373

[3] Mohamed Z, Roshan TM (2009) Human *Plasmodium knowlesi*: An emerging infection presented with severe thrombocytopenia. International Medical Journal 16: 307–310.

[4] Barber BE, William T, Grigg MJ, Menon J, Auburn S, et al (2013) A prospective comparative study of knowlesi, falciparum, and vivax malaria in Sabah, Malaysia: High proportion with severe disease from Plasmodium knowlesi and Plasmodium vivax but no mortality with early referral and artesunate therapy. Clin Infect Dis 56: 383–397. 23087389 10.1093/cid/cis902

[5] Singh B, Daneshvar C (2010) *Plasmodium knowlesi* malaria in Malaysia. Med J Malaysia 65: 223–230. 21939162

[6] William T, Rahman HA, Jelip J, Ibrahim MY, Menon J, et al (2013) Increasing incidence of *Plasmodium knowlesi* malaria following control of *P. falciparum* and *P. vivax* malaria in Sabah, Malaysia. PLoS Negl Trop Dis 7 (1) e2026. 23359830 10.1371/journal.pntd.0002026 PMC3554533

[7] Anonymous (2013) Imported malaria cases and deaths, United Kingdom: 1993–2012. Public Health England

[8] Ramaswami A, Pisharam JK, Aung H, Ghazala K, Maboud K, et al (2013) Co-incidental *Plasmodium knowlesi* and Mucormycosis infections presenting with acute kidney injury and lower gastrointestinal bleeding. Am J Case Rep 14: 103–105. 23826445 10.12659/AJCR.883879 PMC3700492

[9] Khim N, Siv S, Kim S, Mueller T, Fleischmann E, et al (2011) *Plasmodium knowlesi* infection in humans, Cambodia, 2007–2010. Emerg Infect Dis 17: 1900–1902. 22000366 10.3201/eid1710.110355 PMC3310675

[10] Figtree M, Lee R, Bain L, Kennedy T, Mackertich S, et al (2010) *Plasmodium knowlesi* in human, Indonesian Borneo. Emerg Infect Dis 16: 672–674. 20350383 10.3201/eid1604.091624 PMC3321967

[11] Sulistyaningsih E, Fitri LE, Löscher T, Berens-Riha N (2010) Diagnostic difficulties with *Plasmodium knowlesi* infection in humans. Emerg Infect Dis 16: 1033. 20507769 10.3201/eid1606.100022 PMC3086231

[12] Sermwittayawong N, Singh B, Nishibuchi M, Sawangjaroen N, Vuddhakul V (2012) Human *Plasmodium knowlesi* infection in Ranong province, southwestern border of Thailand. Malar J 11: 36. 22313518 10.1186/1475-2875-11-36 PMC3293766

[13] Zhu H, Li J, Zheng H (2006) Human natural infection of *Plasmodium knowlesi* . Chin J Parasitol Parasit Dis 24: 70. 16866152

[14] Jiang N, Chang Q, Sun X, Lu H, Yin J, et al (2010) Co-infections with *Plasmodium knowlesi* and other malaria parasites, Myanmar. Emerg Infect Dis 16: 1476–1478. 20735938 10.3201/eid1609.100339 PMC3294981

[15] Tyagi RK, Das MK, Singh SS, Sharma YD (2013) Discordance in drug resistance-associated mutation patterns in marker genes of *Plasmodium falciparum* and *Plasmodium knowlesi* during coinfections. J Antimicrob Chemother 68: 1081–1088. 23292346 10.1093/jac/dks508

[16] Luchavez J, Espino F, Curameng P, Espina R, Bell D, et al (2008) Human Infections with *Plasmodium knowlesi*, the Philippines. Emerg Infect Dis 14: 811–813. 18439369 10.3201/eid1405.071407 PMC2600254

[17] Ennis JG, Teal AE, Habura A, Madison-Antenucci S, Keithly JS, et al (2009) Simian malaria in a U.S. traveler - New York, 2008. Morb Mortal Weekly Rep 58: 229–232. 19282815

[18] Jeslyn WP, Huat TC, Vernon L, Irene LM, Sung LK, et al (2011) Molecular epidemiological investigation of *Plasmodium knowlesi* in humans and macaques in Singapore. Vector Borne Zoonotic Dis 11: 131–135. 20586605 10.1089/vbz.2010.0024 PMC3033207

[19] Ng OT, Ooi EE, Lee CC, Lee PJ, Ng LC, et al (2008) Naturally acquired human *Plasmodium knowlesi* infection, Singapore. Emerg Infect Dis 14: 814. 18439370 10.3201/eid1405.070863 PMC2600232

[20] Ong CWM, Lee SY, Koh WH, Ooi EE, Tambyah PA (2009) Case report: Monkey malaria in humans: A diagnostic dilemma with conflicting laboratory data. Am J Trop Med Hyg 80: 927–928. 19478250

[21] Putaporntip C, Hongsrimuang T, Seethamchai S, Kobasa T, Limkittikul K, et al (2009) Differential prevalence of *Plasmodium* infections and cryptic *Plasmodium knowlesi* malaria in humans in Thailand. J Infect Dis 199: 1143–1150. 19284284 10.1086/597414 PMC8817623

[22] Berry A, Iriart X, Wilhelm N, Valentin A, Cassaing S, et al (2011) Imported *Plasmodium knowlesi* malaria in a French tourist returning from Thailand. Am J Trop Med Hyg 84: 535–538. 21460005 10.4269/ajtmh.2011.10-0622 PMC3062444

[23] Jongwutiwes S, Putaporntip C, Iwasaki T, Sata T, Kanbara H (2004) Naturally acquired *Plasmodium knowlesi* malaria in human, Thailand. Emerg Infect Dis 10: 2211. 15663864 10.3201/eid1012.040293 PMC3323387

[24] Jongwutiwes S, Buppan P, Kosuvin R, Seethamchai S, Pattanawong U, et al (2011) *Plasmodium knowlesi* malaria in humans and macaques, Thailand. Emerg Infect Dis 17: 1799–1806. 22000348 10.3201/eid1710.110349 PMC3310673

[25] Van den Eede P, Van HN, Van Overmeir C, Vythilingam I, Duc TN, et al (2009) Human *Plasmodium knowlesi* infections in young children in central Vietnam. Malar J 8: 249. 19878553 10.1186/1475-2875-8-249 PMC2773789

[26] Marchand RP, Culleton R, Maeno Y, Quang NT, Nakazawa S (2011) Co-infections of *Plasmodium knowlesi*, *P. falciparum*, and *P. vivax* among humans and *Anopheles dirus* mosquitoes, Southern Vietnam. Emerg Infect Dis 17: 1232–1239. 21762577 10.3201/eid1707.101551 PMC3381379

[27] Joveen-Neoh WF, Chong KL, Wong CM, Lau TY (2011) Incidence of malaria in the interior division of Sabah, Malaysian Borneo, based on nested PCR. J Parasitol Res 2011: 104284. 22013506 10.1155/2011/104284 PMC3195446

[28] Cox-Singh J, Davis TM, Lee KS, Shamsul SS, Matusop A, et al (2008) *Plasmodium knowlesi* malaria in humans is widely distributed and potentially life threatening. Clin Infect Dis 46: 165–171. 18171245 10.1086/524888 PMC2533694

[29] Vythilingam I, Noorazian YM, Huat TC, Jiram AI, Yusri YM, et al (2008) *Plasmodium knowlesi* in humans, macaques and mosquitoes in peninsular Malaysia. Parasit Vectors 1: 26. 18710577 10.1186/1756-3305-1-26 PMC2531168

[30] Kantele A, Marti H, Felger I, Muller D, Jokiranta TS (2008) Monkey malaria in a European traveler returning from Malaysia. Emerg Infect Dis 14: 1434–1436. 18760013 10.3201/eid1409.080170 PMC2603100

[31] Bronner U, Divis PC, Farnert A, Singh B (2009) Swedish traveller with *Plasmodium knowlesi* malaria after visiting Malaysian Borneo. Malar J 8: 15. 19146706 10.1186/1475-2875-8-15 PMC2634766

[32] Lee KS, Cox-Singh J, Singh B (2009) Morphological features and differential counts of *Plasmodium knowlesi* parasites in naturally acquired human infections. Malar J 8: 73. 19383118 10.1186/1475-2875-8-73 PMC2676309

[33] Daneshvar C, Davis TM, Cox-Singh J, Rafa'ee MZ, Zakaria SK, et al (2009) Clinical and laboratory features of human *Plasmodium knowlesi* infection. Clin Infect Dis 49: 852–860. 19635025 10.1086/605439 PMC2843824

[34] van Hellemond JJ, Rutten M, Koelewijn R, Zeeman AM, Verweij JJ, et al (2009) Human *Plasmodium knowlesi* infection detected by rapid diagnostic tests for malaria. Emerg Infect Dis 15: 1478–1480. 19788819 10.3201/eid1509.090358 PMC2819855

[35] Hoosen A, Shaw MT (2011) *Plasmodium knowlesi* in a traveller returning to New Zealand. Travel Med Infect Dis 9: 144–148. 21481643 10.1016/j.tmaid.2011.03.002

[36] Barber BE, William T, Jikal M, Jilip J, Dhararaj P, et al (2011) *Plasmodium knowlesi* malaria in children. Emerg Infect Dis 17: 814–820. 21529389 10.3201/eid1705.101489 PMC3321776

[37] Link L, Bart A, Verhaar N, van Gool T, Pronk M, et al (2012) Molecular detection of *Plasmodium knowlesi* in a Dutch traveler by real-time PCR. J Clin Microbiol 50: 2523–2524. 22573596 10.1128/JCM.06859-11 PMC3405625

[38] Anderios F, Mohamed Z, Ratnam S, Ibrahim MY, Awang TAM (2008) Detection of malaria parasites in Sabah by nested polymerase chain reaction: A focus of naturally acquired *Plasmodium knowlesi* infections. Sains Malaysiana 37: 137–141.

[39] Lee C, Adeeba K, Freigang G (2010) Human *Plasmodium knowlesi* infections in Klang Valley, Peninsula Malaysia: a case series. Med J Malaysia 65: 63. 21265252

[40] Lau YL, Tan LH, Chin LC, Fong MY, Noraishah MA-A, et al (2011) *Plasmodium knowlesi* reinfection in human. Emerg Infect Dis 17: 1314. 21762601 10.3201/eid1707.101295 PMC3381378

[41] Naing DKS, Anderios F, Zaw L (2011) Geographic and ethnic distribution of *P. knowlesi* infection in Sabah, Malaysia. Int J Collab Res Internal Med Public Health 5: 391–400.

[42] Barber BE, William T, Dhararaj P, Anderios F, Grigg MJ, et al (2012) Epidemiology of *Plasmodium knowlesi* malaria in north-east Sabah, Malaysia: family clusters and wide age distribution. Malar J 11: 401. 23216947 10.1186/1475-2875-11-401 PMC3528466

[43] Singh B, Daneshvar C (2013) Human infections and detection of *Plasmodium knowlesi* . Clin Microbiol Rev 26: 165–184. 23554413 10.1128/CMR.00079-12 PMC3623376

[44] Garnham PCC (1966) Malaria parasites and other Haemosporidia. Oxford, UK: Blackwell Scientific Publications. 1144 p.

[45] Barber BE, William T, Grigg MJ, Yeo TW, Anstey NM (2013) Limitations of microscopy to differentiate Plasmodium species in a region co-endemic for Plasmodium falciparum, Plasmodium vivax and Plasmodium knowlesi. Malar J 12: 8 doi: 10.1186/1475-2875-12-8 23294844 10.1186/1475-2875-12-8 PMC3544591

[46] Barber BE, William T, Grigg MJ, Piera K, Yeo TW, et al (2013) Evaluation of the sensitivity of a pLDH-based and an aldolase-based Rapid Diagnostic Test for diagnosis of uncomplicated and severe malaria caused by PCR-confirmed Plasmodium knowlesi, Plasmodium falciparum, and Plasmodium vivax. J Clin Microbiol 51: 1118–1123. 23345297 10.1128/JCM.03285-12 PMC3666806

[47] Imwong M, Tanomsing N, Pukrittayakamee S, Day N, White N, et al (2009) Spurious amplification of a *Plasmodium vivax* smallsubunit RNA gene by use of primers currently used to detect *P. knowlesi* . J Clin Microbiol 47: 4173–4175. 19812279 10.1128/JCM.00811-09 PMC2786678

[48] Coatney GR, Collins WE, Warren M, Contacos PG (1971) The Primate Malarias. Atlanta, GA: US Center for Disease Control.

[49] Chin W, Contacos PG, Collins WE, Jeter MH, Alpert E (1968) Experimental Mosquito-Transmission of *Plasmodium Knowlesi* to Man and Monkey. Am J Trop Med Hyg 17: 355. 4385130 10.4269/ajtmh.1968.17.355

[50] Brady OJ, Gething PW, Bhatt S, Messina JP, Brownstein JS, et al (2012) Refining the Global Spatial Limits of Dengue Virus Transmission by Evidence-Based Consensus. PLoS Negl Trop Dis 6 (8) e1760. 22880140 10.1371/journal.pntd.0001760 PMC3413714

[51] WHO (2012) World Malaria Report 2012. Geneva: World Health Organization. 195 p.

[52] Gething PW, Elyazar IRF, Moyes CL, Smith DL, Battle KE, et al (2012) A long neglected world malaria map: *Plasmodium vivax* endemicity in 2010. PLoS Negl Trop Dis 6: e1814. 22970336 10.1371/journal.pntd.0001814 PMC3435256

[53] Gething PW, Patil AP, Smith DL, Guerra CA, Elyazar IRF, et al (2011) A new world malaria map: *Plasmodium falciparum* endemicity in 2010. Malar J 10: 378. 22185615 10.1186/1475-2875-10-378 PMC3274487

[54] IUCN (International Union for Conservation of Nature (2008) Macaca range maps. In: IUCN Red List of Threatened Species. Version 2013.2. Available: http://www.iucnredlist.org/

[55] Collins WE, Contacos PG, Chin W (1978) Infection of the squirrel monkey *Saimiri sciureus*, with *Plasmodium knowlesi* . Trans R Soc Trop Med Hyg 72: 662–663. 104412 10.1016/0035-9203(78)90030-5

[56] Sinka ME, Bangs MJ, Manguin S, Chareonviriyaphap T, Patil AP, et al (2011) The dominant *Anopheles* vectors of human malaria in the Asia-Pacific region: occurrence data, distribution maps and bionomic precis. Parasit Vectors 4. 10.1186/1756-3305-4-89 PMC3127851 21612587

[57] Fielding AH, Bell JF (1997) A review of the methods for the assessment of prediction errors in conservation presence/absence models. Environ Conserv 24: 38–49.

[58] Lambrecht F, Dunn F, Eyles D (1961) Isolation of *Plasmodium knowlesi* from Philippine macaques. 10.1038/1911117a0 13758508

[59] Wharton RH, Eyles DE (1961) *Anopheles hackeri*, a vector of *Plasmodium knowlesi* in Malaya. Science 134: 279–280. 13784726 10.1126/science.134.3474.279

[60] Collins WE, Contacos PG, Guinn EG (1967) Studies on the transmission of simian malarias. II. Transmission of the H strain of *Plasmodium knowlesi* by *Anopheles balabacensis balabacensis* . J Parasitol 53: 841–844. 6035726

[61] Collins W, Contacos P, Skinner J, Guinn E (1971) Studies on the transmission of simian malaria. IV. Further studies on the transmission of *Plasmodium knowlesi* by *Anopheles balabacensis balabacensis* mosquitoes. J Parasitol 57: 961–966. 5002525

[62] Rao D, Chandra K, Devi K (2013) Endemic Animals of Andaman and Nicobar Islands. Kolkata. 182 p.

[63] Sivakumar K (2010) Impact of the Tsunami (December, 2004) on the Long Tailed Macaque of Nicobar Islands, India. Hystrix Italian Journal of Mammalogy 21: 35–42.

[64] Tanizaki R, Ujiie M, Kato Y, Iwagami M, Hashimoto A, et al (2013) First case of *Plasmodium knowlesi* infection in a Japanese traveller returning from Malaysia. Malar J 12: 128. 23587117 10.1186/1475-2875-12-128 PMC3637542

[65] Ta Tang T-H, Salas A, Ali-Tammam M, del Carmen Martinez M, Lanza M, et al (2010) First case of detection of *Plasmodium knowlesi* in Spain by Real Time PCR in a traveller from Southeast Asia. Malar J 9. 10.1186/1475-2875-9-219 PMC2921078 20663184

[66] Kuo MC, T.T C, C.W C, Tsai FW, Ji DD (2009) A case report of simian malaria, *Plasmodium knowlesi*, in a Taiwanese traveller from Plawan island, the Philippines. Taiwan Epid Bull 25: 167–178.

[67] Conlan JV, Sripa B, Attwood S, Newton PN (2011) A review of parasitic zoonoses in a changing Southeast Asia. Vet Parasitol 182: 22–40. 21846580 10.1016/j.vetpar.2011.07.013

[68] Roos C, Thanh VN, Walter L, Nadler T (2007) Molecular systematics of Indochinese primates. Vietn J Primatol 1: 41–53.

[69] Gumert MD, Fuentes A, Jones-Engel L (2011) Monkeys on the edge: ecology and management of long-tailed macaques and their interface with humans. Cambridge, UK: Cambridge University Press. 360 p.

[70] Jones-Engel L, Engel GA, Schillaci MA, Pacheco M, Escalante A (2007) Malarial monkeys: reservoir for zoonotic infection? Am J Primatol 69: 40–41.

[71] Heinsohn T (2003) Animal translocation: long-term human influences on the vertebrate zoogeography of Australasia (natural dispersal versus ethnophoresy). Aust Zool 32: 351–376.

[72] Jones-Engel L, Schillaci M, Engel G, Paputungan U, Froehlich J (2006) Characterizing primate pet ownership in Sulawesi: implications for disease transmission. In: Paterson JD, Wallis J, editors. Commensalism and conflict: the human-primate interface. Norman, OK: American Society of Primatologists pp. 196–221.

[73] Long J (2003) Introduced mammals of the world: Their history, distribution and influence. Collingwood, Australia: CSIRO Publishing. 612 p.

[74] Wong CL, Ni IH (2000) Population dynamics of the feral macaques in the Kowloon Hills of Hong Kong. Am J Primatol 50: 53–66. 10588435 10.1002/(SICI)1098-2345(200001)50:1<53::AID-AJP5>3.0.CO;2-A

[75] Shek C-T (2011) Management of nuisance macaques in Hong Kong. In: Gumert MD, Fuentes A, Jones-Engel L, editors. Monkeys on the Edge: Ecology and Management of Long-Tailed Macaques and their Interface with Humans. United Kingdon: Cambridge University Press. pp. 297–301.

[76] Hamada Y, Urasopon N, Hadi I, Malaivijitnond S (2006) Body size and proportions and pelage color of free-ranging Macaca mulatta from a zone of hybridization in northeastern Thailand. International Journal of Primatology 27: 497–513.

[77] Fooden J (1997) Tail length variation in *Macaca fascicularis* and *M. mulatta* . Primates 38: 221–231.

[78] Tosi AJ, Morales JC, Melnick DJ (2002) Y-chromosome and mitochondrial markers in Macaca fascicularis indicate introgression with Indochinese M-mulatta and a biogeographic barrier in the Isthmus of Kra. International Journal of Primatology 23: 161–178.

[79] Bonhomme M, Cuartero S, Blancher A, Crouau-roy B (2009) Assessing Natural Introgression in 2 Biomedical Model Species, the Rhesus Macaque (Macaca mulatta) and the Long-Tailed Macaque (Macaca fascicularis). Journal of Heredity 100: 158–169. 18974398 10.1093/jhered/esn093

[80] Osada N, Uno Y, Mineta K, Kameoka Y, Takahashi I, et al (2010) Ancient genome-wide admixture extends beyond the current hybrid zone between Macaca fascicularis and M-mulatta. Mol Ecol 19: 2884–2895. 20579289 10.1111/j.1365-294X.2010.04687.x

[81] Huffman MA, Satou M, Kawai S, Maeno Y, Kawamoto Y, et al (2013) New perspectives on the transmission of malaria between macaques and humans: The case of Vietnam. Folia Primatol (Basel) 84: 288–289.

[82] Collins WE (2012) *Plasmodium knowlesi*: a malaria parasite of monkeys and humans. Annu Rev Entomol 57: 107–121. 22149265 10.1146/annurev-ento-121510-133540

[83] Wheatley BP (1980) Malaria as a possible selective factor in the speciation of macaques. J Mammal 61: 307–311. 7462867

[84] Garnham PCC (1963) Distribution of simian malaria parasites in various hosts. J Parasitol 49: 905–911. 14084194

[85] Kemp NJ, Burnett JB (2003) Final report: A biodiversity risk assessment and recommendations for risk management of long-tailed macaques (*Macaca fascicularis*) in New Guinea. Indo-Pacific Conservation Alliance. 1–116 p.

[86] Lee KS, Divis PC, Zakaria SK, Matusop A, Julin RA, et al (2011) *Plasmodium knowlesi*: reservoir hosts and tracking the emergence in humans and macaques. PLoS Pathog 7: e1002015. 21490952 10.1371/journal.ppat.1002015 PMC3072369

[87] Seethamchai S, Putaporntip C, Malaivijitnond S, Cui LW, Jongwutiwes S (2008) Malaria and *Hepatocystis* species in wild macaques, Southern Thailand. Am J Trop Med Hyg 78: 646–653. 18385364

[88] Putaporntip C, Jongwutiwes S, Thongaree S, Seethamchai S, Grynberg P, et al (2010) Ecology of malaria parasites infecting Southeast Asian macaques: evidence from cytochrome b sequences. Mol Ecol 19: 3466–3476. 20646216 10.1111/j.1365-294X.2010.04756.x PMC2921002

[89] Tan CH, Vythilingam I, Matusop A, Chan ST, Singh B (2008) Bionomics of *Anopheles latens* in Kapit, Sarawak, Malaysian Borneo in relation to the transmission of zoonotic simian malaria parasite *Plasmodium knowlesi* . Malar J 7: 52. 18377652 10.1186/1475-2875-7-52 PMC2292735

[90] Vythilingam I, Tan CH, Asmad M, Chan ST, Lee KS, et al (2006) Natural transmission of *Plasmodium knowlesi* to humans by *Anopheles latens* in Sarawak, Malaysia. Trans R Soc Trop Med Hyg 100: 1087–1088. 16725166 10.1016/j.trstmh.2006.02.006

[91] Jiram AI, Vythilingam I, NoorAzian YM, Yusof YM, Azahari AH, et al (2012) Entomologic investigation of *Plasmodium knowlesi* vectors in Kuala Lipis, Pahang, Malaysia. Malar J 11: 213. 22727041 10.1186/1475-2875-11-213 PMC3476358

