# Supplementary figures and images for: Defining the Geographical Range of the Plasmodium knowlesi Reservoir
Source: PLoS Negl Trop Dis. 2014 Mar 27;8(3):e2780. doi: 10.1371/journal.pntd.0002780 (PMC3967999; doi:10.1371/journal.pntd.0002780)

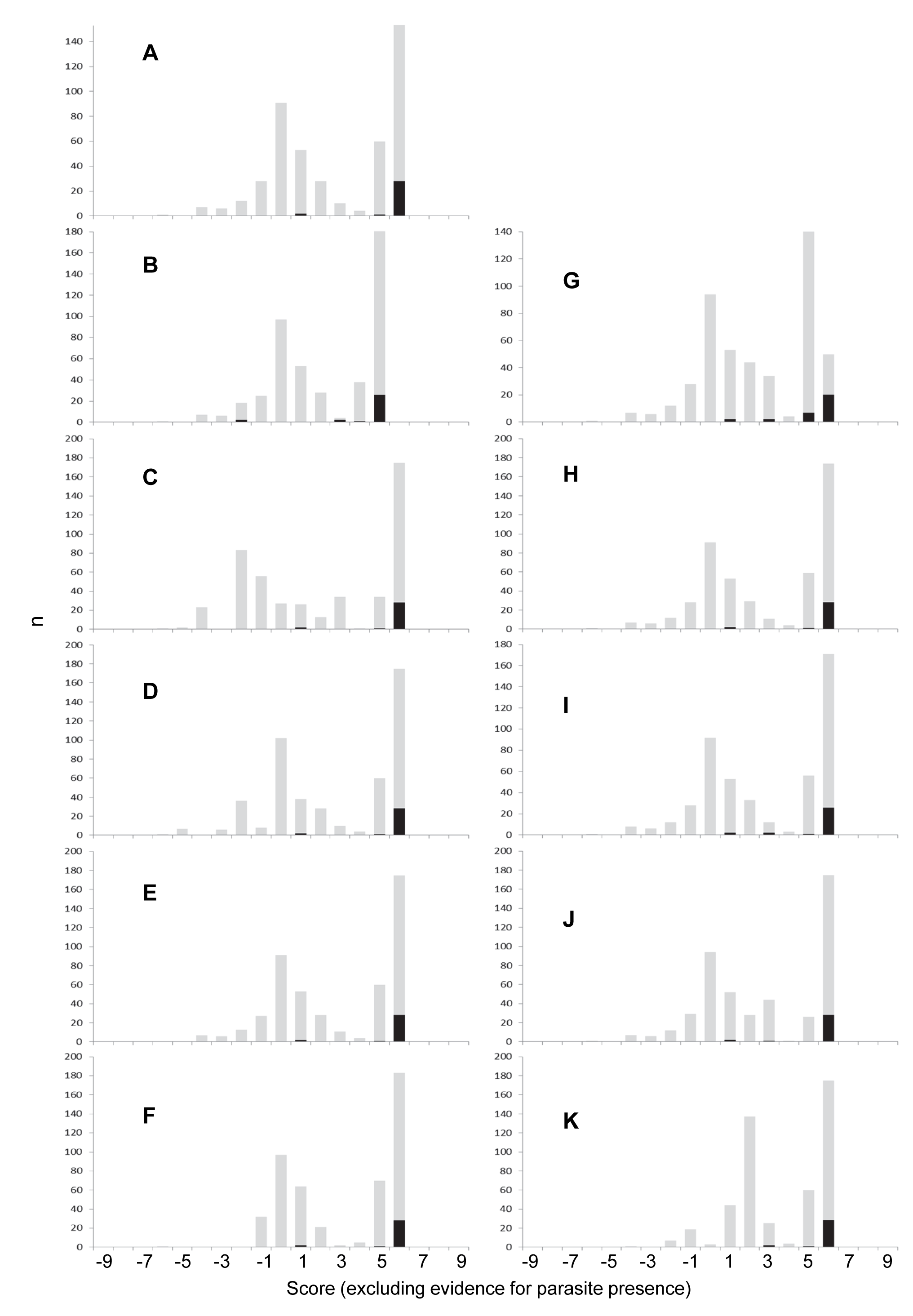

Supplement: Figure S1 — A figure showing histograms of the scores generated each time the scoring system was adjusted. Subnational areas with confirmed cases of knowlesi malaria in either humans or macaques are marked in black and all other areas are light grey. Panel A shows the scores when evidence of parasite presence is excluded. Panels B–L show the scores generated when a second individual evidence class is excluded: B) Leucosphyrus vectors excluded; C) other sylvatic vectors excluded; D) other human vectors excluded; E) combined vector range excluded; F) other human malarias excluded; G) the natural range of M. fasciularis excluded; H) the natural range of M. nemestrina excluded; I) introduced M. fascicularis and M. nemestrina populations excluded; J) M. leonina excluded; K) combined monkey range excluded. (TIF) [file pntd.0002780.s001.tif]

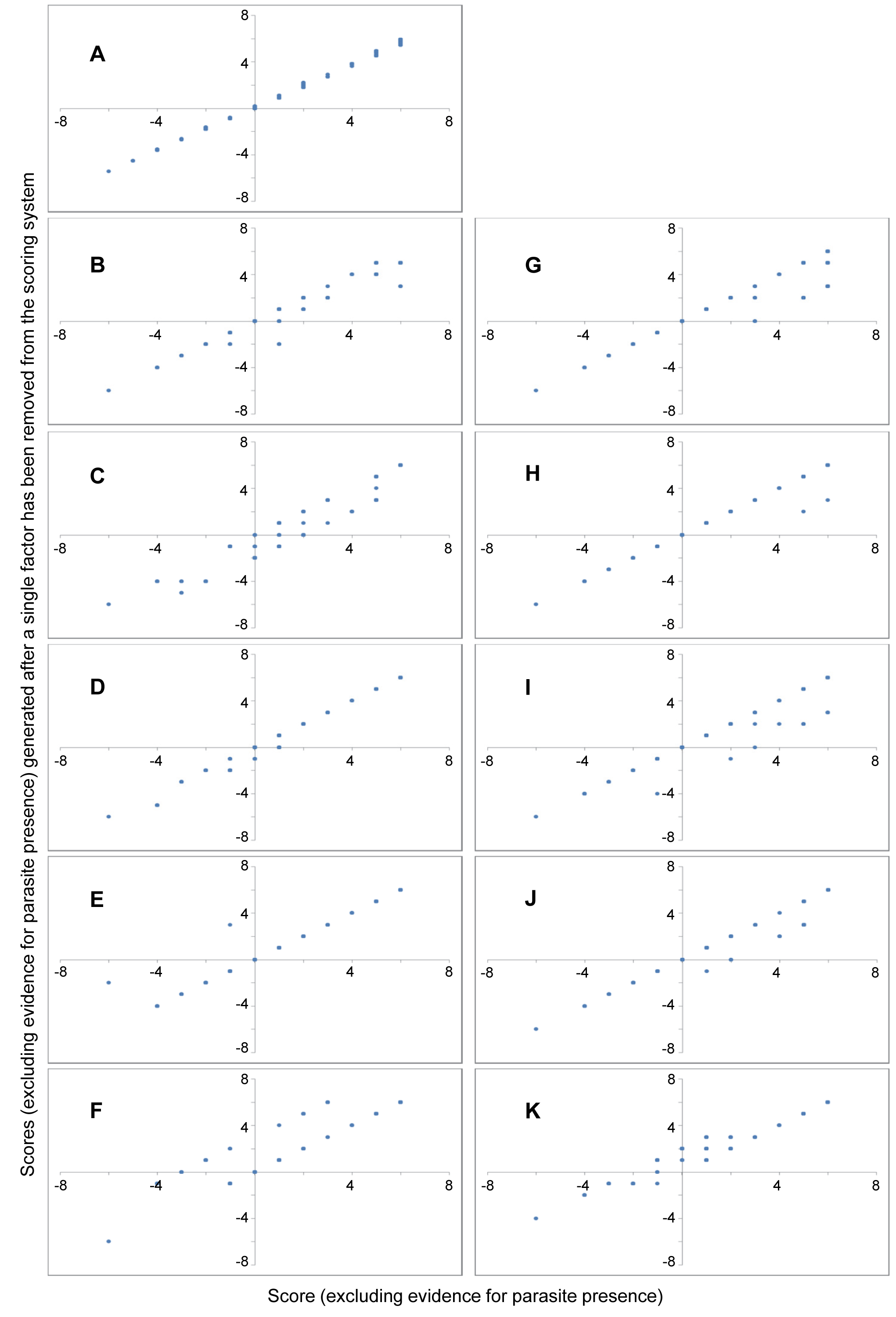

Supplement: Figure S2 — A figure showing ranked scores generated when individual factors were excluded. Each graph shows the ranked scores when evidence of parasite presence is excluded (the x axis) against the ranked scores when the score is adjusted as follows: A) the mean score obtained across all exclusions (B–L); B) Leucosphyrus vectors excluded; C) other sylvatic vectors excluded; D) other human vectors excluded; E) combined vector range excluded; F) other human malarias excluded; G) the natural range of M. fasciularis excluded; H) the natural range of M. nemestrina excluded; I) introduced M. fascicularis and M. nemestrina populations excluded; J) M. leonina excluded; K) combined monkey range excluded. (TIF) [file pntd.0002780.s002.tif]
